# Neurotoxicity Comparison of Two Types of Local Anaesthetics: Amide-Bupivacaine versus Ester-Procaine

**DOI:** 10.1038/srep45316

**Published:** 2017-03-24

**Authors:** Xu-jiao Yu, Wei Zhao, Yu-jie Li, Feng-xian Li, Zhong-jie Liu, Hua-li Xu, Lu-ying Lai, Rui Xu, Shi-yuan Xu

**Affiliations:** 1 Zhujiang Hospital, Southern Medical University, Guangzhou City, China

## Abstract

Local anaesthetics (LAs) may lead to neurological complications, but the underlying mechanism is still unclear. Many neurotoxicity research studies have examined different LAs, but none have comprehensively explored the distinct mechanisms of neurotoxicity caused by amide- (bupivacaine) and ester- (procaine) type LAs. Here, based on a CCK8 assay, LDH assay, Rhod-2-AM and JC-1 staining, 2′,7′-dichlorohy-drofluorescein diacetate and dihydroethidium probes, an alkaline comet assay, and apoptosis assay, we show that both bupivacaine and procaine significantly induce mitochondrial calcium overload and a decline in the mitochondrial membrane potential as well as overproduction of ROS, DNA damage and apoptosis (P < 0.05). There were no significant differences in mitochondrial injury and apoptosis between the bupivacaine and procaine subgroups (P > 0.05). However, to our surprise, the superoxide anionic level after treatment with bupivacaine, which leads to more severe DNA damage, was higher than the level after treatment with procaine, while procaine produced more peroxidation than bupivacaine. Some of these results were also affirmed in dorsal root ganglia neurons of C57 mice. The differences in the superoxidation and peroxidation induced by these agents suggest that different types of LAs may cause neurotoxicity via different pathways. We can target more accurate treatment based on their different mechanisms of neurotoxicity.

Local anaesthetics (LAs), which are divided into amide and ester types based on their chemical structure, are frequently used in subarachnoid blocks, epidural blocks, local infiltration, peripheral nerve blocks and postoperative analgesia. However, numerous studies have indicated that LAs are cytotoxic at certain concentrations in many types of cells. That is, the toxicity depends not only on the concentration but also on the cell type. In addition, there are several case reports of cell damage (muscle, cartilage) at clinically relevant concentrations^1^,^2^,^3^,^4^,^5^,^6^,^7^,^8^. Although toxicity caused by clinically relevant concentrations is rare, it may produce severe complications when it occurs. Specifically, transient neurological symptoms (approximately happen in 1/3 patients), persistent lumbosacral neuropathy (a risk of 1/1300–1/200) and, the most severe complication, cauda equine syndrome (incidence from 1/1000–1/10000) after spinal anaesthesia have attracted attention for many years^9^,^10^,^11^. A recent multicentre prospective survey on the incidence of major complications in patients undergoing regional anaesthesia (RA) in China revealed the incidence of major RA complications, including paraplegia (n = 1; 0.09/10,000) and cauda equina syndrome (n = 2; 0.19/10,000)^12^. For some of these complications, LA neurotoxicity is thought to be the principle elements^13^. However, the exact underlying mechanisms of LA neurotoxicity are still not fully understood.

Several aspects are involved in LA neurotoxicity, including apoptosis^14^,^15^,^16^,^17^, the inhibition of voltage-dependent calcium channels^3^,^18^, mitochondrial dysfunction^19^,^20^,^21^,^22^, endoplasmic reticulum calcium depletion^23^, and DNA damage^24^. Our previous research has revealed that bupivacaine-activated ROS production^25^,^26^ and autophagy^24^ are also involved. Perez-Castro *et al*. compared the cytotoxicity of six frequently used LAs in SH-SY5Y cells and observed that bupivacaine and lidocaine can trigger cell apoptosis. Meanwhile, Werdehausen *et al*. compared eight local anaesthetics (two of the ester and six of the amide type). They investigated the apoptotic potency resulting from different LAs at the LD_50_ concentration and finally discovered that the neurotoxicity of the LAs correlated with the lipid solubility but was independent of the chemical class (ester or amide type). Afterward, Arnaud Malet *et al*. assessed the neurotoxicity of different LAs on SH-SY5Y cells using the WST-1 method^17^,^27^,^28^. Mete evaluated the neurotoxic effects of four LAs on the mouse neuroblastoma NB2a cell line^11^. Some scholars have also compared the neurotoxicity of different structures of LAs^29^. Nevertheless, few studies have comprehensively examined the distinct neurotoxicity mechanisms of amide- and ester-type drugs.

Due to the different types of LAs, there may be differences in efficacy, duration of action, and drug toxicity. Large concentrations, continuous application and long exposure time can cause neurotoxicity^11^. The mechanism of local anaesthetic neurotoxicity has been examined in multiple reports, but there has been no direct comparison of the mechanisms of neurotoxicity for different types of anaesthetic drugs. Whether differences in drug types lead to differences in their neurotoxicity mechanisms is unknown.

In this study, our first aim was to explore the neurotoxic effects of bupivacaine and procaine on SH-SY5Y cells, which represent amide LAs and ester LAs, respectively. Subsequently, determination of DNA damage, mitochondrial dysfunction, reactive oxygen species (ROS) production, and cellular apoptosis were used to compare the underlying differences in the effects of these two types of LAs on SH-SY5Y cells. In addition, since a primary site of action of LAs administered neuraxially is the dorsal root ganglia (DRG) neurons^13^, some of the effects were also confirmed in DRG neurons of C57 mice.

## Results

### Effects of bupivacaine (Bup) and procaine (Pro) on SH-SY5Y viability and cytotoxicity

**Figure 1:**
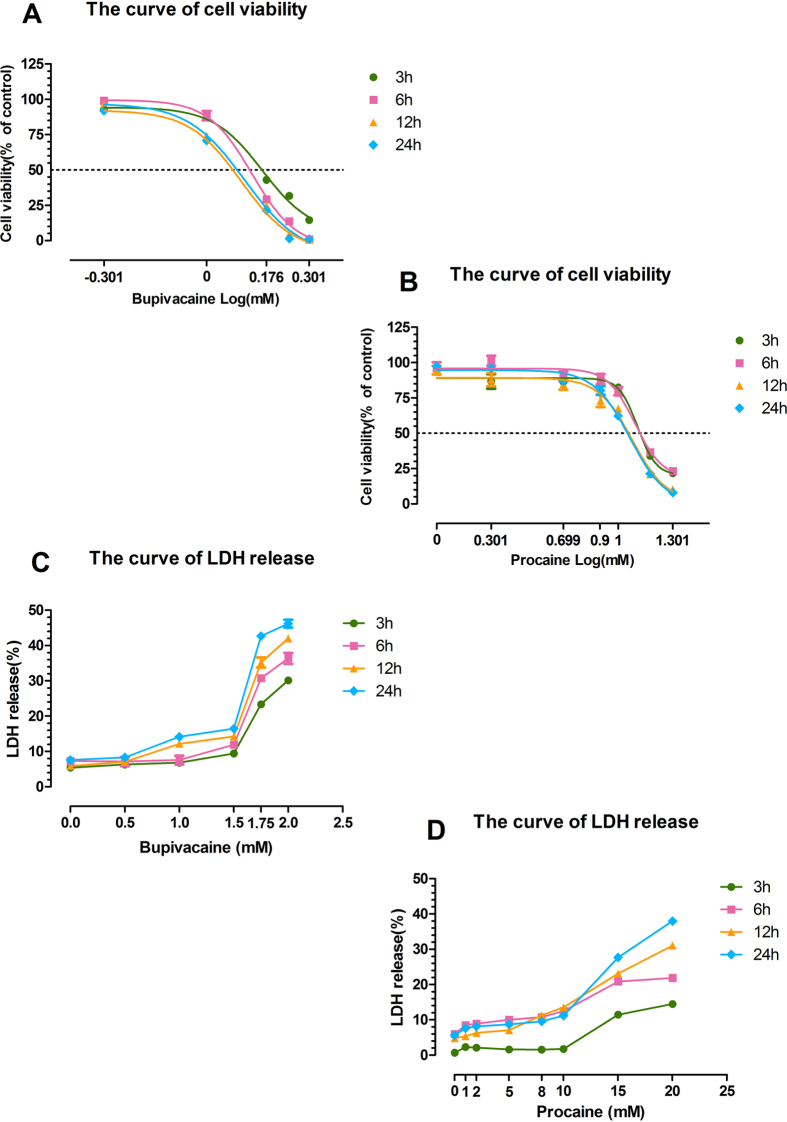
Effects of bupivacaine and procaine on SH-SY5Y cells measured using the CCK-8 cell viability assay and LDH cytotoxicity assay. Drugs were prepared at the following concentrations: 0.5, 1.0, 1.5, 1.75, and 2.0 mM for bupivacaine and 1, 2, 5, 8, 10, 15, and 20 mM for procaine. Dose-response and time-response curves of the cell viability of cells treated with bupivacaine (**A**) and procaine (**B**) were measured while cells were recovering in a regular medium at 6 h, 12 h and 24 h after a 3-h exposure. Curves were generated by nonlinear regression using the following equation: Viability (*x*) = Bottom + (Top-Bottom)/[1 + 10^(LogIC50-X)*HillSlope^], where x is the Log(concentration), the Viability (*x*) is the remaining viability (in % of the untreated control values), and IC50 is the concentration at which a particular LA had 50% of its maximal killing capacity. Data were analysed using GraphPad Prism 5, GraphPad Software, Inc, San Diego, CA. Shown in the figure are the means ± SD from 3 independent experiments performed in quintuplets. Cytotoxicity was assessed by LDH release after exposure to anaesthetics. High concentrations of the two anaesthetics increased LDH release. The curves showing LDH release when treated by bupivacaine (**C**) and procaine (**D**) at different time points are shown.

After being exposed to Bup (0.5, 1.0, 1.5, 1.75, and 2.0 mM) and Pro (1, 2, 5, 8, 10, 15, and 20 mM) for 3 h and recovering in fresh basic media for 0 h, 3 h, 9 h, and 21 h (the total time is 3 h, 6 h, 12 h, 24 h, respectively), SH-SY5Y cell viability was reduced in a dose-dependent fashion at the four time points (Fig. 1A,B). The LD_50_ values of Bup at the four time points were approximately 1.458, 1.359, 1.293, and 1.263 mM, while the values of Pro were approximately 12.79, 12.38, 11.96, and 11.58 mM. Thus, 1.3 mM Bup and 12 mM Pro were selected to be used in the cell injury model in the other studies. Meanwhile, Bup (Fig. 1C) and Pro (Fig. 1D) significantly increased neurotoxicity at high concentrations, as shown by increased lactate dehydrogenase (LDH) release.

### Bupivacaine and procaine induced mitochondrial dysfunction in SH-SY5Y cells and DRG neurons

**Figure 2:**
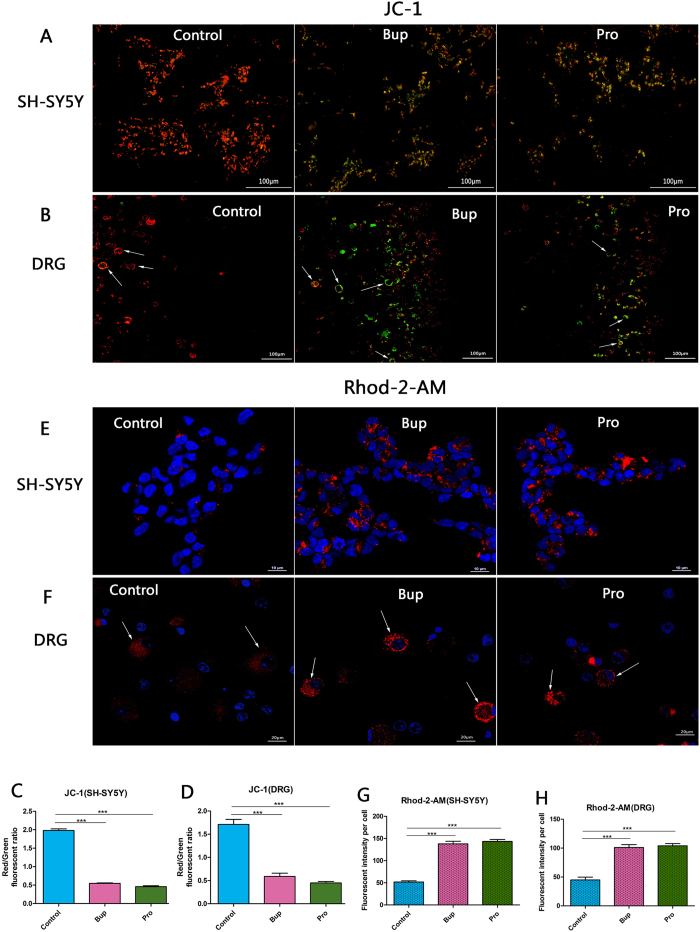
Bupivacaine and procaine induced mitochondrial dysfunction in SH-SY5Y cells (**A**,**E**) and DRG neurons (**B**,**F**). The change in mitochondrial membrane permeability (MMP) was tested by JC-1 staining (**A**,**B**); the control group cells had strong J-aggregation and appeared red, while the majority of the cells in the two anaesthetic groups were stained green due to low Δψm. Mitochondrial calcium overload was assessed by Rhod-2-AM (**E**,**F**). Mitochondrial calcium levels were compared between the bupivacaine, procaine, and control groups of SH-SY5Y cells and DRG neurons. A red/green analysis of the fluorescence intensity per cell indicated that there were no differences between the two drugs (**C**,**D**). *P < 0.05, **P < 0.01, ***P < 0.001.

Bup and Pro treatment of the SH-SY5Y cells decreased the mitochondrial membrane potential (MMP) level as evidenced by the fact that the control group cells presented red or orange, while the majority of the cells in the two anaesthetic groups were stained green (Fig. 2A). The fluorescence intensity was expressed as the red/green fluorescence ratio, and compared with that of the control group, the fluorescence intensities of the treated groups were significantly reduced (Fig. 2C; *P* < 0.001). Similar results were also shown in the DRG neurons (Fig. 2B,D; *P* < 0.001). However, there were no statistically significant differences between the Bup and Pro subgroups (*P* = 0.068). The mitochondrial calcium levels were measured using Rhod-2-AM (Fig. 2E,F). Bup and Pro led to a significant increase in mitochondrial calcium compared to that in untreated cells (Fig. 2G; *P* < 0.001). The interaction of the treated groups did not reach statistical significance (*P* = 0.499). This mitochondrial calcium overload was also affirmed in the DRG neurons (Fig. 2F,H; *P* < 0.001 compared to control). These results suggested that bupivacaine and procaine promoted mitochondrial dysfunction in SH-SY5Y cells and DRG neurons.

### Effects of bupivacaine and procaine on cellular ROS production detected in SHSY5Y cells and DRG neurons

**Figure 3:**
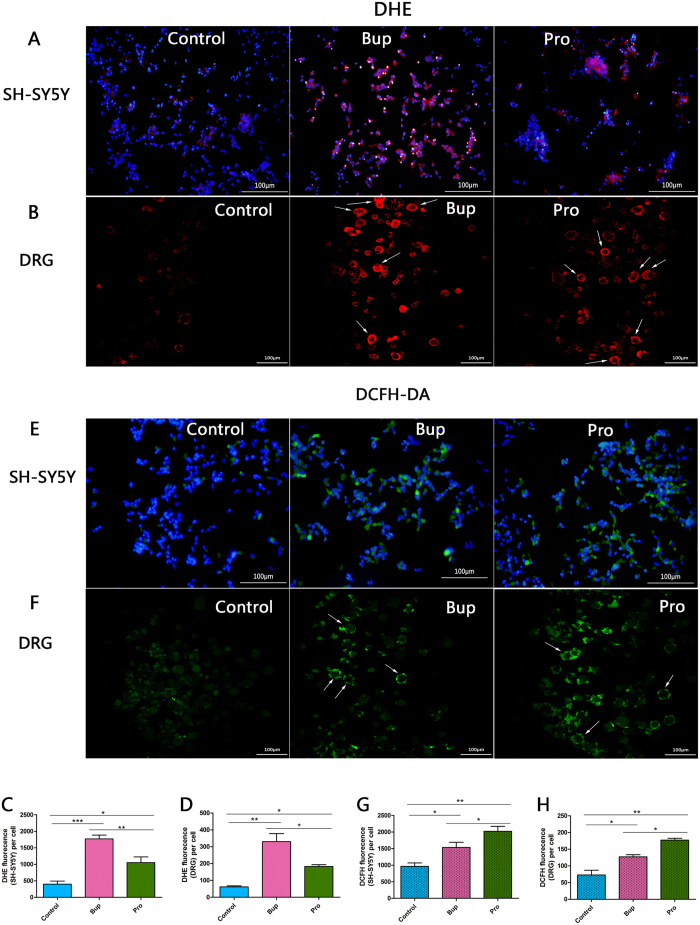
Effects of bupivacaine and procaine on cellular ROS (reactive oxygen species) production detected by dihydroethidium (DHE) staining and dichlorofluorescein diacetate (DCFH-DA) in SH-SY5Y cells (**A**,**E**) and DRG neurons (**B**,**F**). Quantitative analysis of the fluorescence intensity per cell showed that bupivacaine treatment was followed by a rapid burst of ROS, while procaine increased the peroxidation content. *P < 0.05, **P < 0.01, ***P < 0.001.

In the dihydroethidium (DHE) assay, there were significant differences in superoxide anion (O_2_^+ −^) production among all of the groups (Fig. 3A,C; *P* < 0.001 Bup vs control; *P* < 0.05 Pro vs control; *P* < 0.01 Bup vs Pro), and the Bup group showed the highest level of red fluorescence. In the 2′,7′-dichlorohy-drofluorescein diacetate (DCFH-DA) assay, exposure to Bup and Pro resulted in a marked increase in peroxidation generation compared to that observed in the control group (Fig. 3E,G; *P* < 0.05 Bup vs control; *P* < 0.01 Pro vs control; *P* < 0.05 Bup vs Pro). Amazingly, the peroxidation content of the cells in the presence of Pro significantly increased compared to that in the presence of Bup, which presented the highest intensity of green fluorescence. The same effects were also detected in the DRG neurons (Fig. 3B,F). These results demonstrated that bupivacaine and procaine induced the production of ROS, thereby generating oxidative stress. However, this variance from the DCFH-DA and DHE assay may suggest that different types of LAs cause cytotoxicity through different mechanisms.

### An alkaline comet assay and western blotting were used to test bupivacaine- and procaine-induced cell DNA damage

**Figure 4:**
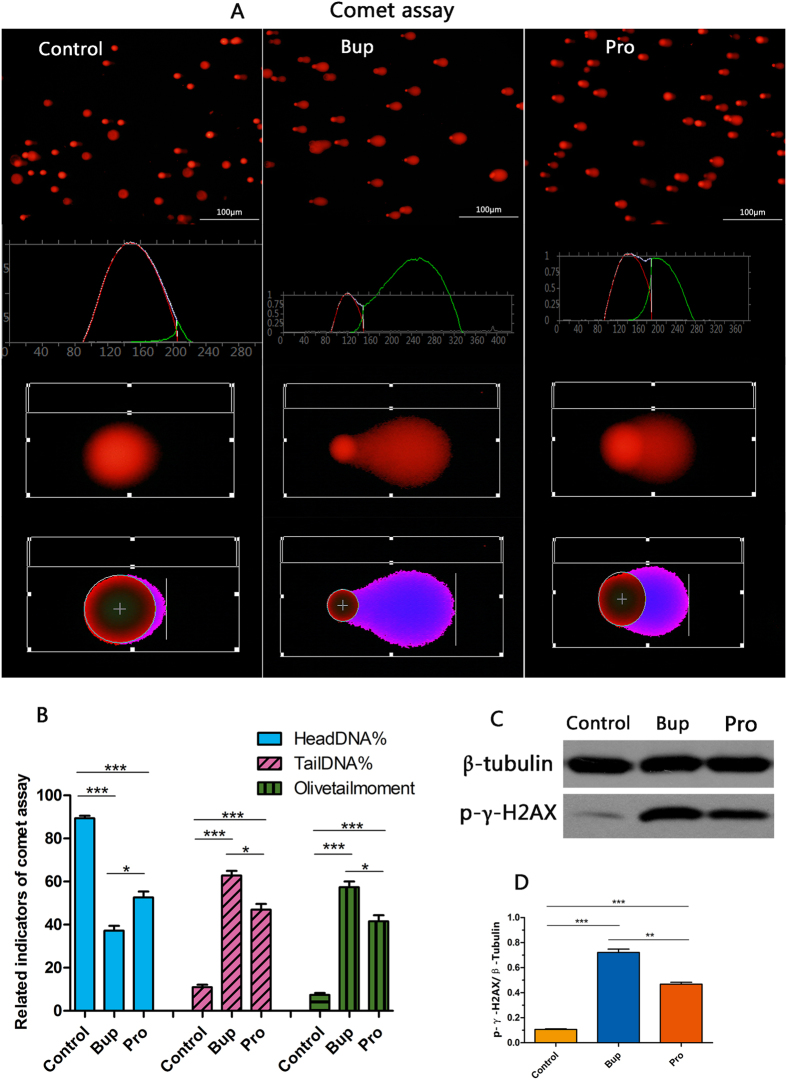
An alkaline comet assay (**A**) and Western blotting (**C**) were used to test bupivacaine- and procaine-induced cell DNA damage. A shows the comet track and the analysis graph of the three groups. Related indicators of the comet assay, including Head DNA%, Tail DNA% and Olive tail moment%, were in accordance with the DNA damage. Bupivacaine and procaine upregulated the expression of p-γ-H2AX. Statistical analysis indicated a significant difference between the two anaesthetics in the induced DNA damage (**B**,**D**). *P < 0.05, **P < 0.01, ***P < 0.001.

The DNA damage of SH-SY5Y cells was examined using an alkaline comet assay and western blotting (Fig. 4). Compared with that of the Pro group, the cell nuclei of the Bup group had longer tails and smaller heads, but the damage in both the Pro and Bup groups was more severe than the damage in the control group (Fig. 4A). Significant differences were found in the three indicators related to DNA damage between the treated samples and controls; the control cells had a higher head DNA% (both *P* < 0.001 compared to that of the Bup and Pro groups). In the Bup- and Pro-treated groups, the tail DNA% and olive tail moment were increased with a decreased head DNA% (*P* < 0.001 compared to that of control group), and the analysis of the two treated groups also reached statistical significance. Consistent with the comet assay, the expression of p-γ-H2AX in the Bup and Pro groups showed a robust upregulation (Fig. 4C; *P* < 0.001) and there were also significant differences between the treated groups (*P* < 0.001). These results demonstrated that bupivacaine and procaine indeed induced DNA damage, and the former agent caused more severe damage (Table 1).

### TUNEL staining and western blotting were performed to study the influence of bupivacaine and procaine on cell apoptosis

**Figure 5:**
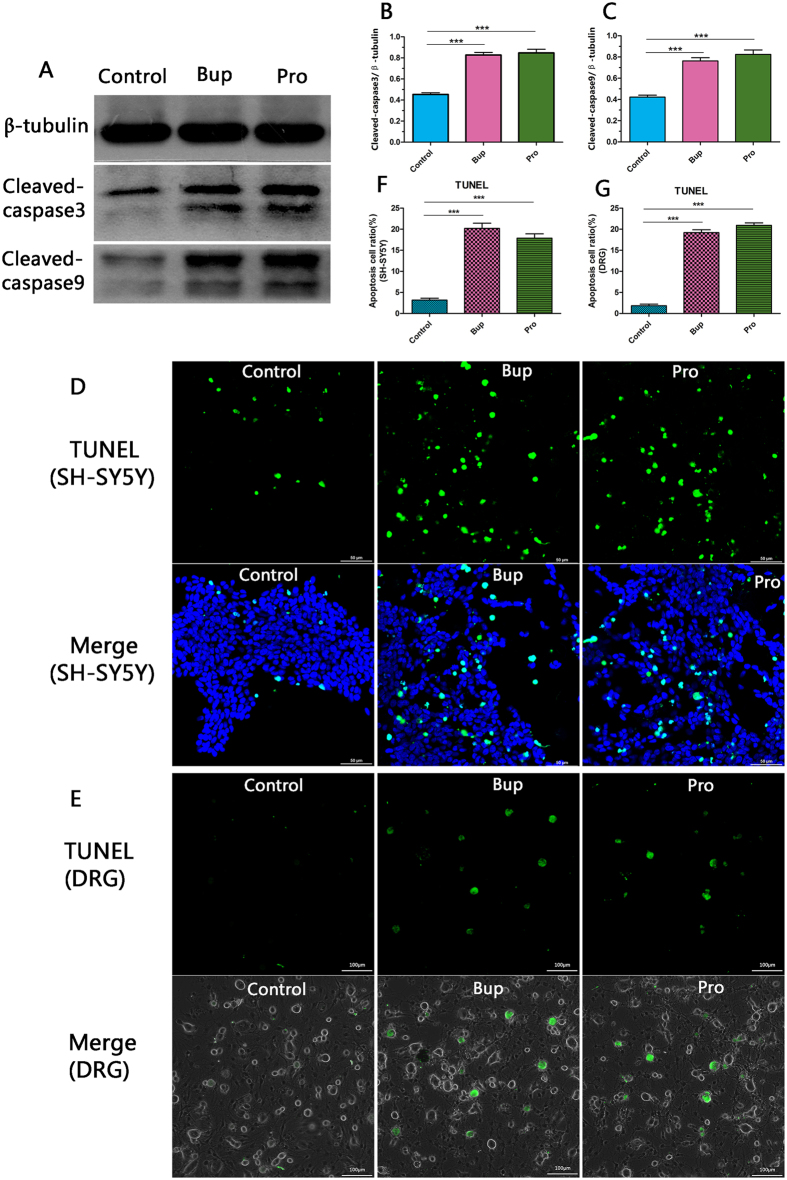
TUNEL staining and western blotting were performed to study the influence of bupivacaine and procaine on cell apoptosis. The drugs promoted SH-SY5Y cell apoptosis (**A**) as shown by the increased expression of cleaved caspase-9 and cleaved caspase-3. The grey value of the protein band was analysed by Image J, and the relative expression of apoptosis was statistically analysed. Each value was presented as the mean ± SD of three independent experiments. These results were confirmed in SH-SY5Y cells and DRG neurons by TUNEL staining (**D**,**E**). Each experiment was performed at least 3 times. ***P < 0.001.

Stimulation of SH-SY5Y cells with Bup and Pro led to a dramatic increase in apoptosis protein as evidenced by the cleaved caspase-9 and cleaved caspase-3 noted with western blotting (Fig. 5A,B,C; *P* < 0.001 Bup vs control; *P* < 0.001 Pro vs control). This result was confirmed in the SH-SY5Y cells and DRG neurons using the TUNEL assay (Fig. 5D,E). However, there was still no significant difference between the Bup and Pro subgroups (cleaved caspase-3, *P* = 0.67; cleaved caspase-9, *P* = 0.296; TUNEL, *P* = 0.07 and *P* = 0.12). These data indicated that bupivacaine and procaine may simultaneously cause cell apoptosis.

## Discussion

Our study demonstrated that the cell viability of SH-SY5Y cells declined and neurotoxicity increased in a concentration-dependent manner after treatment with bupivacaine and procaine (Fig. 1). A comparison of the LD_50_ values of the two LAs resulted in the following order of neurotoxic tendency: bupivacaine > procaine. These results corresponded with the results of previous studies^17^,^30^. The underlying mechanisms may involve mitochondrial dysfunction, including mitochondrial calcium overload and mitochondrial membrane potential reduction (Fig. 2), overproduction of ROS (Fig. 3), DNA damage (Fig. 4), and neuronal apoptosis (Fig. 5). SH-SY5Y cells of the human neuroblastoma cell line are widely used in cytotoxic research^23^,^24^,^27^,^28^. Because the cytotoxic response is similar to that of human primary neuronal cultures^16^. A primary site of action of LAs administered neuraxially is the DRG neurons, which have also been used as a model for neurotoxicity^13^; thus, these cells were chosen as our cell model.

Bupivacaine, an amide-type LA, is commonly used clinically is also frequently studied in neurotoxicology testing^17^,^24^,^28^. Procaine is a classic short-acting ester-type local anaesthetic and has been in use because of its low toxicity^17^,^27^,^30^. However, the detailed mechanism of LA neurotoxicity has still not been clarified. In the past, Kalichman and colleagues assessed the neural toxicity and relative motor nerve conduction blockade of two amide LAs (etidocaine and lidocaine) and two ester LAs (chloroprocaine and procaine) in rat sciatic nerves. Their findings suggested that both the toxic effects and the conduction block were not consistent with their proposal that ester agents are more likely than other LAs to cause nerve injury; they also found a perfect correlation between nerve blocking concentrations and toxic concentrations^31^. Based on these findings, subsequent comparative research reports on the different LAs and their effects in diverse cell types have been published. Werdehausen and partners compared eight LAs and calculated the concentration of the LD_50_ to assess early apoptosis. They discovered that ester- and amide-type LAs are equally neurotoxic, and the neurotoxicity of eight LAs (including bupivacaine and procaine) correlated with the octanol/buffer coefficients but were independent of the structure (ester or amide)^17^. Meanwhile, Perez-Castro and colleagues compared the cytotoxicity of LAs in human neuronal cells and detected apoptosis activity, depolarization and the carbachol-stimulated intracellular Ca^2+^-responses of lidocaine and bupivacaine. They observed that lidocaine and bupivacaine could activate caspase-3/-7, leading to apoptosis, but their patterns of activation were different^27^. Because the diffusion of LAs was affected by multiple factors, such as the concentration gradient, the affinity for lipids, and their protein-binding ability and pKa, Malet compared the neurotoxicity of seven LAs in undifferentiated SH-SY5Y cells, taking the physicochemical properties of pKa and lipid water partition coefficient (LWPC) into account^28^. However, the shortcoming of the previous studies is that none of them comprehensively explored the distinct mechanisms of neurotoxicity of amide- and ester-type drugs, which underscores the significance of our study. Here, we tested whether differences in drug types lead to differences in their neurotoxicity mechanisms.

Our results proved that bupivacaine and procaine could induce mitochondrial dysfunction, an ROS burst, DNA damage, and neural apoptosis in human neuroblastoma cell line SH-SY5Y cells at the concentration that resulted in the half-maximal neurotoxic effects (LD_50_). There was no significant difference between bupivacaine and procaine in the induced mitochondrial dysfunction and neuronal apoptosis (Figs 2 and 5). Nevertheless, to our surprise, the superoxide anionic level after treatment with bupivacaine, which led to more severe DNA damage, was higher than that after treatment with procaine (Figs 3A–D and 4). In contrast, procaine induced more peroxidase generation than bupivacaine (Figs 3E–H and 4). Most of these results were also confirmed in DRG neurons (Figs 2, 3 and 5). What caused these phenomena?

Previous finding suggested that LAs in clinically relevant doses could evoke apoptosis of rabbit AF cells, involving, at least in part, the mitochondrial pathway^32^. Calcium is well known to be an important second messenger in the regulation of many cellular physiological functions, and calcium overload is harmful to mitochondrial function. Hung and colleagues observed the relationship between bupivacaine toxicity and extracellular calcium and described that increasing CaCl_2_ content prolonged the nerve block of bupivacaine but resulted in histopathologic changes in the rat sciatic nerve^33^. This result was aligned with the viewpoint of Doan LV that lidocaine cytotoxicity involved other pathways apart from the lidocaine-induced effects on cytosolic calcium responses^13^. T-type calcium channels may be involved in bupivacaine neurotoxicity^34^. Furthermore, LAs increased mitochondrial reactive oxygen species (ROS), caused mitochondrial dysfunction (mitochondrial DNA damage, a decrease in adenosine triphosphate and mitochondrial protein levels), dysregulated mitochondrial Ca^2+^ signalling, elevated mitochondrial oxidant stress, and caused nerve membrane solubilization resulting in irreversible neural injury and apoptosis^35^,^36^. Bupivacaine exerts its neurotoxic effects in SH-SY5Y cells by generating excess ROS and activating autophagy via regulation of the PI3K (i.e., PIK3CB and PIK3R2) signalling pathway^24^. Apoptosis and necrosis were mediated by oxidative stress, a condition in which ROS, such as superoxide (O2-), hydrogen peroxide (H_2_O_2_), hydroxyl radical (SOH), and nitric oxide (SNO), are overproduced^37^,^38^. Similarly, our previous study demonstrated that bupivacaine induced ROS overproduction and cell apoptosis in SH-SY5Y cells via the activation of an AMPK-dependent pathway^25^.

Therefore, on the basis of our results, we boldly speculate that after neuronal cells are induced by bupivacaine and procaine, mitochondrial calcium overload is the essential effect that plays a crucial role in the mechanisms of the oxidative stress-induced increases in mitochondrial reactive oxygen species (ROS) via the activation of an AMPK-dependent pathway, including the calcium**-**stimulated increase in the metabolic rate, cytochrome *c* dissociation, peroxidation, and superoxidation, leading to a change in the permeability of the mitochondrial membranes, and then CI^−^ influx into mitochondria causing mitochondrial depolarization and a decline in the mitochondrial membrane potential. Total cellular ROS are also increased, generating oxidative stress and resulting in DNA damage, apoptosis, and even cellular death^19^,^39^,^40^. Because bupivacaine and procaine are two different types of LAs, neurotoxicity may have resulted from the production of different ROS (Fig. 3). While bupivacaine primarily induced superoxidation leading to severe DNA damage, procaine mainly contributed to peroxidation. These differing results were likely related to their anaesthetic types. Procaine was hydrolysed by plasma esterases to nontoxic metabolites and displayed a powerful antioxidant. When significant amounts of procaine enter the circulation, the hydrolysing capacity may be exceeded, and toxicity occurs, thus bringing about lower toxicity. The precise mechanism for this effect awaits further exploration. A large number of studies on the neurotoxicity of LAs have been reported with the nearly consistent conclusion that the neurotoxicities of different LAs are varied. Werdehausen and colleagues compared the apoptosis potency of eight anaesthetics on the basis of the comparison of the LD_50_ values and deemed that LAs induced neuronal apoptosis, and their neurotoxicity was related to their lipid solubility but independent of their chemical class (ester/amide)^17^. Muguruma compared the neurotoxicity of intrathecal bupivacaine, levobupivacaine, and dextrobupivacaine in rats and suggested that the neurotoxicity of these three anaesthetics was similar^29^. However, because the neurotoxicity mechanisms of LAs are complicated and involve multiple factors, comparing only a single aspect of their neurotoxicity mechanism was inadequate. Are there any differences in the other aspects of the neurotoxicity mechanism ? Hence, we designed a comparative study of the neurotoxicity mechanisms of LAs. Our results were in keeping with Werdehausen and indicated that there was no significant difference in the apoptosis of neuronal cells caused by bupivacaine and procaine. Nevertheless, there was a statistically significant difference in the bupivacaine- and procaine-induced ROS burst and DNA damage. Were these differences caused by their different types ? More evidence is required to clarify this issue. In addition, DNA damage is an early event that initiates the appropriate mechanisms to activate the enzymes for DNA repairment^41^. If repairment failed, it will cause cell damage and even cell death. Our comet assay detect early DNA damage, while TUNEL assay can be used to detect the late DNA damage in the late stage of apoptosis. Our results showed that there may be no statistical difference in late DNA damage between two kinds of LAs. Although there are differences in early DNA damage, but the reduction of differences in the late of DNA damage may prompt us: whether the mechanism is different for the two drugs to evoke DNA damage repair? This is also worth for further study.

At present, some preventive measures have been applied in clinical practice to avoid LA toxicity, such as using biologically “smart” hydrogel microparticles (MPs) composed of biodegradable polymers to limit the adverse analgesic effects through the controlled release of bupivacaine^42^. The use of lipid emulsions has reduced the cardiac toxicity of LAs after the occurrence of neurological, cardiovascular, or intramuscular symptoms of systemic intoxication^43^. However, few effective measures are specifically aimed at LA neurotoxicity. There is an urgent need for us to understand the underlying neurotoxicity mechanisms of different types of LAs to target treatment.

## Conclusions

In summary, our study demonstrated that bupivacaine (a long-acting amide LA) and procaine (a short-acting ester LA) exerted neurotoxicity in a dose-dependent manner. Their related mechanisms may be mitochondrial dysfunction (mitochondrial calcium overload and mitochondrial membrane potential reduction), overproduction of ROS, DNA damage, and neuronal apoptosis. The differences in superoxidation and peroxidation induced by bupivacaine and procaine remind us that different types of LAs (ester or amide) exert neurotoxicity via different pathways. However, our experiment may also have some limitations: for example, each type of LA is distinctive, their related mechanisms are not well understood, and the *in vitro* experimental model may be relatively poor. We aimed to mimic the clinical model of LA neurotoxicity so that we can find out the underlying mechanisms. Further validation of these results needs to be demonstrated in future studies. Based on our experiments, effective strategies to reduce the superoxidation and peroxidation caused by bupivacaine and procaine may contribute to discovery of differential interventions. However, support for this concept through further investigation is needed.

## Methods

### Animals

Healthy neonatal male C57 mice (8~10 d) were purchased from the Laboratory Animal Centre of Southern Medical University (Guangzhou, China). All of the animal procedures were approved by the Institutional Animal Care and Use Committee at the Southern Medical University and were performed in accordance with the latest National Institutes of Health Guide for the Care and Use of Laboratory Animals.

### Cell culture

Undifferentiated human neuroblastoma cell line SH-SY5Y cells (SH-SY5Y, SCSP-5014), purchased from the Cell Bank of Shanghai Institute for Biological Science, Chinese Academy of Science, were cultured with a DMEM/F12 medium containing 10% foetal bovine serum with 1% penicillin-streptomycin (Gibco, Invitrogen Life Technology, Carlsbad, CA, USA) at 37 °C and 5% CO_2_ atmosphere. The cells were maintained in fresh medium every 2 days and passaged every 5 days. Experiments were performed with 80% confluent cells from passages 20–30.

### DRG isolation and culture

Before the mice were killed, coverslips (12 mm diameter) were cleaned with 95% ethanol and shortly burnt. PDL (0.5 mg/ml) was added to the coverslips, and they were maintained at room temperature (RT) for 40 min. Deionized water was used to wash the coverslips, and then laminin (10 μg/ml) was added before maintaining the coverslips at RT for 45 min. Then, 8~10-day-old male mice were killed using ether; the DRG were isolated under a dissecting microscope within 1 h, pooled into culture medium on ice, and then incubated in an enzyme solution (collagenase 342 U/ml, dispase II 3.8 U/ml) (Gibco, Invitrogen, Carlsbad, USA) at 37 °C for 30 min until the end of digestion. The supernatant was collected, and 15% BSA was used to filter the large cells. The suspension was centrifuged at 300 g for 3 min. The pellet was resuspended in the culture medium and plated on precoated coverslips. After a 24 h culture, the neurons of the DRG with neurite growth were used for the experiments.

### Cell viability and LDH cytotoxicity assay

The LAs were dissolved in ultrapure water to make the stock solutions. Subsequently, the LAs were diluted in DMEM/F12 (Gibco, Carlsbad, CA, USA) for use to ensure a steady physiological pH. DMEM/F12-LA solutions were prepared at the following concentrations: 0.5, 1.0, 1.5, 1.75, and 2.0 mM/L for bupivacaine and 1, 2, 5, 8, 10, 15, and 20 mM/L for procaine (both from Sigma-Aldrich, Saint Louis, MO, USA). Addition of LAs did not change the pH value of the medium (7.38, range 7.35–7.42).

The SH-SY5Y cells were seeded at a density of 1.2 × 10^4^ cells per well in a 96-well cell culture plate. Cultured cells were exposed to experimental solutions of bupivacaine (Bup) and procaine (Pro) for 3 h and allowed to recover in a fresh basic medium for 0 h, 3 h, 9 h, and 21 h after drug removal (the total time is 3 h, 6 h, 12 h, 24 h, respectively). At the end of the LA treatment, medium (containing detached cells) was collected from each well and centrifuged for 2 min at 1000 rpm. Each pellet was resuspended and mixed in a previous well so that both the adherent cells and the detached cells were measured. Cell viability was determined using a cell counting kit-8 (CCK-8) assay according to the manufacturer’s protocol (Dojindo, Japan). All of the experiments were repeated at least in triplicate, and for each experiment, the concentrations of the tested LAs were performed in quintuplets. The viability of the control group without LA treatment was set to 100%, and the other groups were normalized to the corresponding control values.

The cytotoxic effects of the LAs on the cell membrane integrity were determined by measuring the activity of LDH using an LDH assay kit (Beyotime, Shanghai, China). Briefly, after exposure to Bup and Pro, the cell culture supernatant was incubated with diagnostic reagents in the LDH kit according to the manufacturer’s instructions. The activity of LDH was calculated as the following equation after detection at 490 nm using a microplate spectrophotometer (SpectraMax M5, California, USA): Experimental cytotoxicity% = (Experimental release – background group)/(maximum release - background group)^44^.

### Alkaline comet assay

The comet assay is a sensitive method to assess DNA damage by quantifying the amount of denatured DNA fragments that migrate out of the cell nuclei during electrophoresis. The cells were immediately mixed with low melting point agarose after being exposed to 3 h of Bup or Pro and dispensed into the comet plate. After the cells were frozen solid, normal agarose was distributed in the culture plate, and the plate was then immersed in cold lysis buffer for 1.5 h at 4 °C. Electrophoresis was performed in an alkali buffer at 30 V, 300 mA for another 30 min, and then the contents were stained with 2.5 g ml^−1^ propidium iodide for 10 min. The images were observed and captured with a fluorescence microscope (Olympus, TH4-200, Japan) at 200x magnification. Fifty randomly selected cells from each group were analysed with the Comet Assay Software Project (CASP-6.0, University of Wroclaw, Poland).

### Western blot analysis

The cells were exposed to Bup and Pro for 3 h to determine the DNA damage, and other cells were allowed to recover in a basic medium for 12 h after 3 h of LA exposure to detect apoptosis. A 30-μg protein sample was loaded per lane, separated by electrophoresis on 12% sodium dodecyl sulphate polyacrylamide gels (SDS-PAGE), and electrotransferred onto polyvinylidene fluoride (PVDF) membranes (Immobilon P, Millipore, Bedford, MA, USA). After blotting, the PVDF membranes were blocked and then immunoblotted overnight at 4 °C with primary antibodies (all from Cell Signalling Technology, Beverly, MA) recognizing cleaved caspase-3 (rabbit, 1:1000; 9661), cleaved caspase-9 (mouse, 1:1000; 9504), phospho-histone H_2_A.X (rabbit, 1:1000; 9718), and β-tubulin (rabbit, 1:1000; 2146), and the membranes were incubated with secondary horseradish peroxidase (HRP). The signal was visualized using ECL reagent. The protein bands were analysed with Image J software (National Institutes of Health, Bethesda, MD, USA).

### Measurement of intracellular ROS

The intracellular ROS generation induced by the LAs was assessed by measuring the fluorescence intensity of DCF and DHE (all from Sigma-Aldrich, Saint Louis, MO, USA). The SH-SY5Y cells and DRG neurons were exposed to Bup and Pro for 3 h and then recovered in regular media for 12 h. Afterwards, the cells were loaded with DCFH-DA and DHE according to the manufacturer’s instructions. Finally, all of the samples were observed using a fluorescence microscope (Olympus, TH4-200, Japan) or confocal microscope and fluorescence-activated cell sorting (FACS; Nikon, TI-FL, Japan) at 200x magnification. The images were analysed using Image-Pro Plus software.

### Assessment of mitochondrial dysfunction

Mitochondrial calcium content was measured using the calcium-sensitive dye Rhod-2-AM (Gibco, Carlsbad, CA, USA). After the SH-SY5Y cells and DRG neurons were treated with Bup and Pro for 3 h and were allowed to recover for 6 h, they were incubated with HBSS-Rhod-2-AM. Before the fluorescence measurements were performed, the cells were washed in indicator-free medium and incubated for a further 30 min to allow for complete deesterification of intracellular AM esters. JC-1, a mitochondrial-specific lipophilic cationic fluorescence probe, was used to monitor the mitochondrial membrane potential (MMP) in the SH-SY5Y cells and DRG neurons. After being treated with Bup and Pro for 3 h and recovering for 24 h, the cells were washed and then maintained in a JC-1 (KeyGEN, Nanjing, China)-containing DMEM/F12 medium. The images were observed and captured with a fluorescence microscope (Olympus, TH4-200, Japan) or FACS (Nikon, TI-FL, Japan). The ratio of red/green JC-1 fluorescence was used as a marker to measure the change in MMP.

### TUNEL assay

The TUNEL assay was conducted to detect apoptosis according to the TUNEL assay kit manufacturer’s instructions (Roche, Mannheim, Germany). Briefly, the SH-SY5Y cells and DRG neurons were treated with Bup and Pro for 3 h and then allowed to recover for 12 h. The test samples were fixed, permeabilizated, and then dyed with the TUNEL reaction mixture (mixture ratio of enzyme solution: label solution = 1:10). The fluorescent signal was measured using FACS (Nikon, TI-FL, Japan). For each group, at least five fields of view were randomly selected to determine the percentage of TUNEL-positive cells among the total cells.

### Statistical analysis

The results are expressed as the mean ± standard deviation (SD). Each experiment was performed at least 3 times. All of the data were analysed using GraphPad Prism 5 (GraphPad Software, San Diego, CA, USA). Cell viability curves were generated by nonlinear regression. The statistical analysis of the quantitative multiple group comparisons was assessed with one-way ANOVA followed by Bonferroni’s post-test. To compare the mean percentages of the relative indicators, repeated-measures analysis of variance (ANOVA), followed by Tukey multiple comparison tests, was used. Otherwise, categorical variables were tested with the nonparametric Kruskal-Wallis test followed by Dunn’s multiple comparisons. Differences between groups with a P-value < 0.05 were considered statistically significant.

## Additional Information

**How to cite this article**: Yu, X.-j. *et al*. Neurotoxicity Comparison of Two Types of Local Anaesthetics: Amide-Bupivacaine versus Ester-Procaine. *Sci. Rep.* **7**, 45316; doi: 10.1038/srep45316 (2017).

**Publisher's note:** Springer Nature remains neutral with regard to jurisdictional claims in published maps and institutional affiliations.

## Figures and Tables

**Table 1:** Differences in the neurotoxicity mechanism of bupivacaine and procaine.

	ROS- superoxidation	ROS- peroxidation	DNA damage
Bup	++	+	++
Pro	+	++	+
Control	−	−	−

The superoxide anionic level after treatment with bupivacaine, which leads to more severe DNA damage, was higher than the level after treatment with procaine, while procaine produced more peroxidation than bupivacaine.
